# MicroBayesAge: a maximum likelihood approach to predict epigenetic age using microarray data

**DOI:** 10.1007/s11357-025-01716-4

**Published:** 2025-05-31

**Authors:** Nicole Nolan, Megan Mitchell, Lajoyce Mboning, Louis-S. Bouchard, Matteo Pellegrini

**Affiliations:** 1 Department of Chemistry and Biochemistry, University of California Los Angeles, Los Angeles, CA 90095 USA; 2 Department of Computational and Systems Biology, University of California Los Angeles, Los Angeles, CA 90095 USA; 3 Department of Molecular, Cell and Developmental Biology, University of California Los Angeles, Los Angeles, CA 90095 USA

**Keywords:** MicroBayesAge, DNA methylation, CpG dinucleotide

## Abstract

Certain epigenetic modifications, such as the methylation of CpG sites, can serve as biomarkers for chronological age. Previously, we introduced the BayesAge frameworks for accurate age prediction through the use of locally weighted scatterplot smoothing (LOWESS) to capture the nonlinear relationship between methylation or gene expression and age, and maximum likelihood estimation (MLE) for bulk bisulfite and RNA sequencing data. Here, we introduce MicroBayesAge, a maximum likelihood framework for age prediction using DNA microarray data that provides less biased age predictions compared to commonly used linear methods. Furthermore, MicroBayesAge enhances prediction accuracy relative to previous versions of BayesAge by subdividing input data into age-specific cohorts and employing a new two-stage process for training and testing. Additionally, we explored the performance of our model for sex-specific age prediction which revealed slight improvements in accuracy for male patients, while no changes were observed for female patients.

**Supplementary information:**

The online version contains supplementary material available at 10.1007/s11357-025-01716-4.

## Introduction

While human genetic sequences remain broadly unchanged over time, the epigenome is highly dynamic and subject to modifications that can be influenced by environmental factors, lifestyle, and aging. Epigenetic modifications to DNA occur over the course of a lifetime, and certain epigenetic changes can be used as a means of determining a person’s age [1]. These epigenetic changes include histone modifications, non-coding RNA interactions, and, most prominently, DNA methylation [2]. In particular, CpG methylation, in which a methyl group is added to the C5 of the cytosine in a CpG dinucleotide, can serve as a robust epigenetic indicator for aging [3]. This process affects gene expression without altering the underlying genetic code and can be influenced by both intrinsic and extrinsic factors. CpG methylation patterns have been widely studied and linked to various biological processes, including cellular differentiation, senescence, and disease progression [4]. Moreover, DNA methylation at specific CpG sites has become a reliable biomarker for estimating both chronological and biological age, concepts that have implications for aging research, forensic science, and personalized medicine.

Different methods of predicting chronological age by analyzing the epigenome have been developed, commonly referred to as “epigenetic clocks” [5–8]. Several of these epigenetic clocks involve DNA methylation analysis using various types of regression algorithms, such as elastic net regression or penalized regression. The development of these clocks has provided valuable tools for aging research. However, the accuracy and applicability of the age predictions can vary depending on the population, the type of tissue analyzed, and the specific algorithm used. There is ongoing research to refine these models to improve their accuracy, reduce biases, and increase their generalizability across different cohorts.

Few methods of age prediction developed to date account for the non-linearity of the relationship between methylation and age [8, 9]. Previous research has shown that, for many CpG sites, methylation changes occur more rapidly at younger ages and more slowly at older ages, suggesting a relationship that is more logarithmic than linear [10]. Recognizing the limitations of existing linear and penalized regression models, we developed an age prediction algorithm, BayesAge 1.0 [11], which predicts age from bisulfite converted sequence data using a combination of locally weighted scatterplot smoothing (LOWESS) regression and maximum likelihood estimation (MLE). BayesAge 1.0 used LOWESS smoothing to account for nonlinear relationships, resulting in less biased age predictions when compared to other commonly used epigenetic clocks that assume a linear relationship. For BayesAge 2.0, we implemented a similar framework as its predecessor to predict transcriptomic age based on gene expression profiles [12]. However, while BayesAge 1.0 and 2.0 were developed to predict the epigenetic and transcriptomic age from bulk bisulfite and RNA sequencing datasets respectively, they are not applicable to microarray technology such as the Illumina 450 K BeadChip platform [13].

Herein, we introduce MicroBayesAge, a Bayesian method for epigenetic age prediction utilizing microarray data. This method leverages CpG sites for age prediction that are most highly correlated with chronological age, as identified via Spearman rank correlation. During the training phase, LOWESS smoothing captures the nonlinear relationship between methylation and age for each selected CpG site, and the variance per CpG site is calculated to create a reference matrix for the predefined age bins. In the prediction phase, the mean and variance are used to fit a normal distribution to each CpG site. The sum of the log probabilities of these highly correlated CpG sites is used to generate a maximum likelihood age distribution, providing an accurate estimate of chronological age. MicroBayesAge offers a streamlined and precise method for epigenetic age prediction to enhance our understanding of the aging process. This innovative approach improves upon previous models by offering robustness to missing data and increased interpretability.

Furthermore, we implement a two-stage Bayesian framework that accurately predicts age for younger people, generating predictions that are, on average, within 1 year of the real chronological age. Finally, we explore the prediction accuracy of our models when the training dataset is divided by sex and find accuracy improvements when predicting male ages but no improvements when predicting female ages.

## Materials and Methods

### Collating Age-Associated DNA Methylation Data

MicroBayesAge is designed to predict the age of a person based on CpG methylation data collected via DNA microarray analysis. To develop and test MicroBayesAge, we utilized a dataset downloaded from the NCBI Gene Expression Omnibus [14]. This dataset comprises publicly available methylome data derived from human blood samples gathered in 19 separate epigenetic studies [15–33]. The data were obtained using the Illumina HumanMethylation450 BeadChip for genome-wide CpG site methylation analysis of DNA samples.

**Fig. 1:**
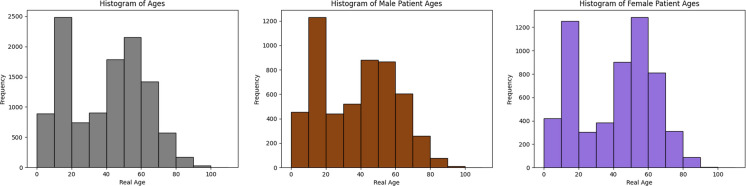
Distribution of real chronological ages for all patients, shown in gray, and for male and female patients in sex-specific datasets, shown in brown and purple respectively

In total, the dataset contains methylomes from over 11,000 patients, each with methylation data of over 200,000 CpG sites, along with their real chronological age and sex. These patients span a wide range of ages, from newborns to centenarians (Fig. 1), providing a broad sample base for the training and validation of MicroBayesAge.

The Spearman rank correlation coefficient between patient age and methylation was calculated for each of the CpG sites. Spearman correlation was used because it is robust for nonlinear trends, such as the logarithmic relationship between methylation and age. We removed from consideration all CpG sites whose Spearman rank correlation had an absolute value of less than 0.60 to focus on the methylation data most relevant for age prediction and increase computational efficiency. This process narrowed the number of CpG sites under consideration to approximately 1500 sites.

To evaluate the MicroBayesAge framework, we employed tenfold cross-validation. The full dataset was split into two groups of samples: testing groups and training groups. The testing groups were created by subdividing the entire dataset into ten mutually exclusive subsets of randomly selected samples, each comprising 10% of the total number of samples. This process randomly generated groups of approximately 1000 test samples from the overall dataset of over 11,000 samples. For each testing group, the remaining 90% of the samples from the full dataset were used as the corresponding training group.

### MLE algorithm

The MLE algorithm employed by MicroBayesAge functions similarly to that of BayesAge 1.0, but it has been updated to handle microarray data. BayesAge 1.0 utilized targeted bisulfite sequencing (TBS-seq) to obtain counts of methylated and unmethylated cytosines at each CpG site. The algorithm assumed that the probability of measuring the observed counts, given the expected methylation level for a particular age, followed a binomial distribution. The probability for a specific CpG site state was computed as a function of the number of reads of cytosines and the number of total reads.

MicroBayesAge, on the other hand, is trained on microarray data from the Illumina HumanMethylation450 BeadChip. In place of counts, signal intensities are converted into a single $\beta$ value that represents the methylation level for a particular CpG site [34]. Without count data, we cannot model the probability as a binomial distribution. Instead, we used the expected methylation levels from the trained model along with the variance to model the probability distribution for a distinct age.

Considering that the variance can change with methylation [35], we chose to estimate the variance by treating samples of the same age as technical replicates. We then plotted the mean methylation level within each age bin against its variance and used LOWESS smoothing to produce a curve of methylation versus variance for each CpG site. We tested $\tau$ values from 0.1 to 1 in intervals of 0.1 and determined that a $\tau$ of 0.7 resulted in predictions with the highest accuracy. For each expected methylation level from the reference matrix, we sampled the predicted variance from this curve and calculated its square root to estimate the standard deviation for a specific age and methylation level.

We then tested both a beta distribution and normal distribution to compute the posterior probability. To fit a beta distribution, we used the mean methylation level from the reference matrix and the variance of a given CpG site to calculate the shape parameters for a distinct age as follows:

$$\alpha =\frac{\mu \left(-{\mu }^{2}+\mu -{\sigma }^{2}\right)}{{\sigma }^{2}} \beta =\frac{\left(\mu -1\right)\left({\mu }^{2}-\mu +{\sigma }^{2}\right)}{{\sigma }^{2}}$$

where $\alpha ,\beta$ is the shape parameters, $\mu$ is the mean methylation level for a given age, ${\sigma }^{2}$ is the variance for a given age and methylation level.

The posterior probability of a given measurement for a particular age was then computed using the beta.pdf function of the scipy.stats Python package. The associated probability for a unique CpG site state is described using the formula below, where ${Pr}_{CpG}$ represents the probability for a given methylation level at a particular age:

$${Pr}_{CpG}\left(x,\alpha ,\beta \right)=\frac{\Gamma \left(\alpha +\beta \right){x}^{\alpha -1}{\left(1-x\right)}^{\beta -1}}{\Gamma \left(\alpha \right)\Gamma \left(\beta \right)}$$

where $\Gamma$ is the gamma function (scipy.special.gamma), $x$ is the measured methylation level for a given subject, $\alpha ,\beta$ is the shape parameters calculated previously.

However, we found that our model performed no differently when calculating the posterior probability using a normal distribution. For simplicity, the posterior probability was modeled using the norm.pdf function of the scipy.stats, as described below [36].

$$\mathcal{N}\left(\mu ,\sigma \right)$$

where $\mu$ is the expected methylation level at the given age, $\sigma$ is the standard deviation for the CpG site at the given age.

To calculate the probability of observing the methylation level measured across all CpG sites for a particular age, we take the logarithmic sum of the probability for each of the top sites. In this manner, the algorithm calculates the likelihood of observing each age from 0 to 100 in a single subject, producing an age likelihood distribution. The maximum likelihood age is predicted as the epigenetic age of the subject.

### Two-stage MicroBayesAge framework

The original BayesAge 1.0 framework consisted of two phases: a training phase and an age prediction phase. In the training phase, a small subset of CpG sites exhibiting the highest absolute value Spearman rank correlation with age was selected for analysis. For MicroBayesAge, extensive optimization testing was performed to determine the optimal number of CpG sites to be used in the training phase to achieve the highest prediction accuracy. After the selection of CpG sites was made, LOWESS regression was applied to model the relationship between age and the methylation levels of the selected CpG sites. We used the LOWESS function from version 0.14.1 of the statsmodels.api Python module [37]. Following further optimization testing, we determined the optimal $\tau$ parameter for the smoothness of the LOWESS fit.

**Fig. 2:**
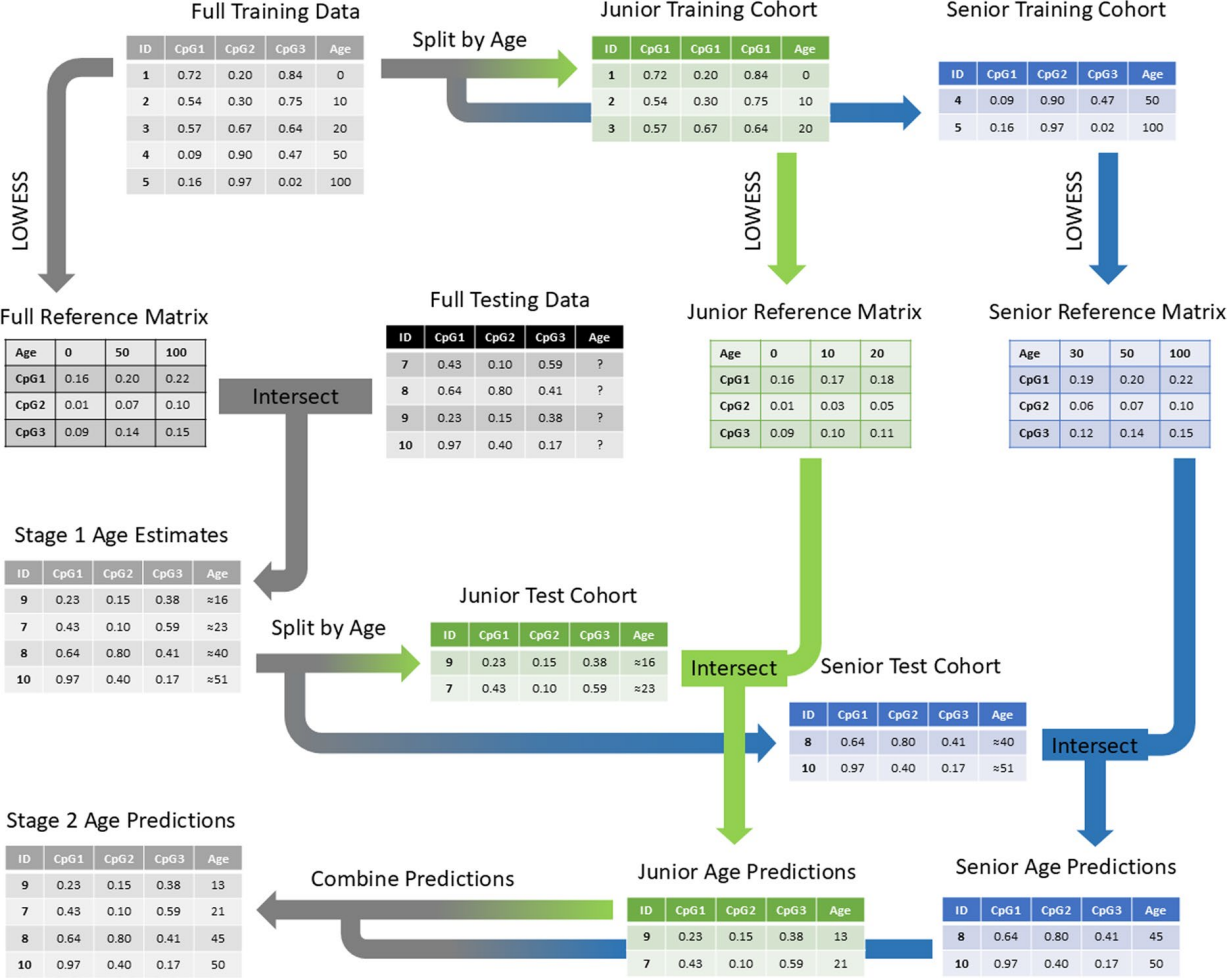
Schematic of the MicroBayesAge framework. The flow of data for all patients is shown in gray. The flow of data for patients aged 25 and younger, the junior cohort, is shown in green. The flow of data for patients aged 26 and older, the senior cohort, is shown in blue

The MicroBayesAge framework utilizes a newly developed methodology that employs a two-stage training phase and a two-stage prediction phase (Fig. 2). In the first stage of the training phase, MicroBayesAge develops its age prediction algorithm using an initial training dataset consisting of CpG site methylation data from individuals with known ages. The framework calculates the Spearman rank correlation coefficient between real age and methylation for each CpG site, using only the available training dataset. The 16 CpG sites that are the most strongly correlated with age are then selected based on these Spearman ranks and the LOWESS function is used to compute the fit for each of the 16 sites.

Age predictions for all samples were analyzed to determine mean absolute error (MAE), root mean squared error (RMSE), and mean bias error (MBE) for each junior cohort maximum age cutoff within the tested range of 10 to 30 years old. This range was chosen to broadly encompass the range of possible ages at which humans could transition from a complex combination of childhood development and aging to purely aging in adulthood with respect to aging-associated DNA methylation [38]. For each cutoff age, a complete set of age predictions was generated and the associated MAE, RMSE, and MBE were calculated for comparison. To choose the optimal cutoff age, we selected the cutoff age with the lowest MBE, since the primary advantage of the BayesAge framework is its low bias compared to other models, rather than its absolute error.

In the second stage of the training phase, the training dataset is split into two cohorts. The junior cohort consists of samples with real ages equal to or less than the optimal cutoff age, and the senior cohort consists of samples with real ages greater than the optimal cutoff age. For each of the two age cohorts, the Spearman rank correlation is recalculated for all CpG sites using only data from that age cohort. Based on the newly calculated Spearman ranks, a new set of the top 16 most correlated CpG sites is identified for each age cohort, and new LOWESS fits are then calculated for the top 16 CpG sites in each cohort.

Once the training phase is complete, the trained model consists of three reference matrices, each comprised of 16 CpG sites and their corresponding methylation levels over possible ages, ranging from 0 to 100 in increments of 1 year. With this trained model, MicroBayesAge can then be used to predict ages for samples with known CpG site methylation levels and unknown real ages.

When given a test dataset with unknown real ages to analyze, a two-stage prediction process is used to predict the age of each sample. For the first stage of the prediction process, the reference matrix developed in the first stage of the training process is intersected with the corresponding CpG sites in the test dataset. MicroBayesAge then employs the maximum likelihood estimation (MLE) to generate initial age estimates based on the intersection of the reference matrix with the CpG site methylation data from the test dataset.

In the second stage of prediction, the algorithm subdivides the input datasets into two different age cohorts based on the initial age estimations. Specifically, a junior cohort is created, consisting of samples with predicted ages of 25 or younger, and a senior cohort is created, comprising samples with predicted ages of 26 or older. For each of these age cohorts, the corresponding reference matrices generated in the second stage of the training phase are intersected with the CpG site methylation data. New age predictions are then calculated separately for each cohort using MLE.

To determine the relative accuracy of the first-stage predictions, which do not attempt to account for any potential differences between age cohorts, compared to the second-stage predictions, we calculated the MAE and RMSE of our age predictions. For both first- and second-stage age predictions, the MAE and RMSE for age predictions of the entire patient population were calculated, as well as the MAE and RMSE for the junior cohort and the senior cohort specifically.

### Sex-specific BayesAge framework

We split the entire training dataset into two groups by sex and similarly split the entire testing dataset into two groups by sex. This resulted in male and female training datasets as well as male and female testing datasets. We then trained and tested the MicroBayesAge framework on the male and female training and testing datasets separately. Comparing the MAE and RMSE calculated for the sex-specific predictions against the MAE and RMSE calculated for the full mixed-sex testing dataset allowed us to determine the impact of sex-specific modeling on the accuracy of age prediction.

### LASSO and elastic net regression comparisons

For comparison testing, we used the Lasso function of the scikit-learn python package. We split our overall dataset into training and testing data for the LASSO regression and elastic net regression using the same random selection method employed to train and test MicroBayesAge. Extensive optimization testing against all training folds was performed. For both LASSO and elastic net, lambda values ranging from 0.01 to 1.00 in increments of 0.01 were tested, and for elastic net, L1 ratios ranging from 0 to 1 in increments of 0.01 were tested. A lambda parameter of 0.01 was found to provide the most accurate age predictions for both LASSO and elastic net. Further, an L1 ratio of 0.72 was found to provide the most accurate age predictions for elastic net.

## Results

### Prediction by age cohort

**Fig. 3:**
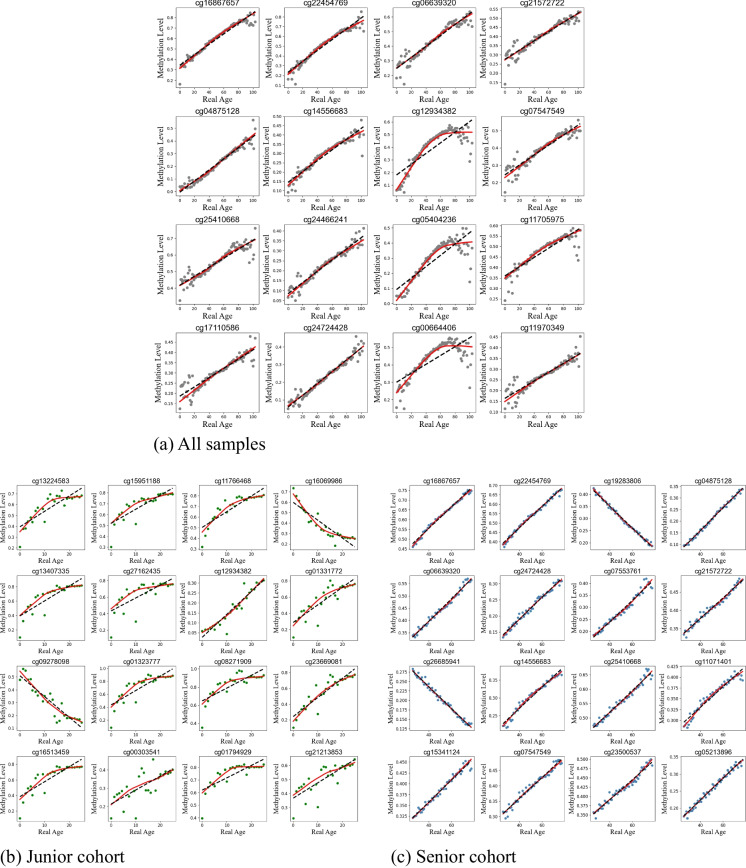
Comparison of LOWESS regression fits, shown in red, with $\tau$ of 0.7 of the relationship between methylation and age for the top 16 most correlated CpG sites. Scatter points are shown in **a** gray for all patients, **b** in green for the junior cohort, and **c** in blue for the senior cohort

After optimization testing, we determined that utilizing only the top 16 CpG sites showing methylation levels with the highest Spearman correlation with age resulted in the highest accuracy (Fig. S1). In the first stage of training, the top sites were selected based on the entire patient dataset (Fig. 3). A LOWESS regression fit of the relationship between methylation and age for these 16 CpG sites was then calculated. These relationships were subsequently used to formulate the first stage of the age predictions for all samples within the validation dataset. The first-stage age predictions for all samples had an MAE of 5.16 years, and RMSE of 6.82, and an MBE of 0.60.

**Fig. 4:**
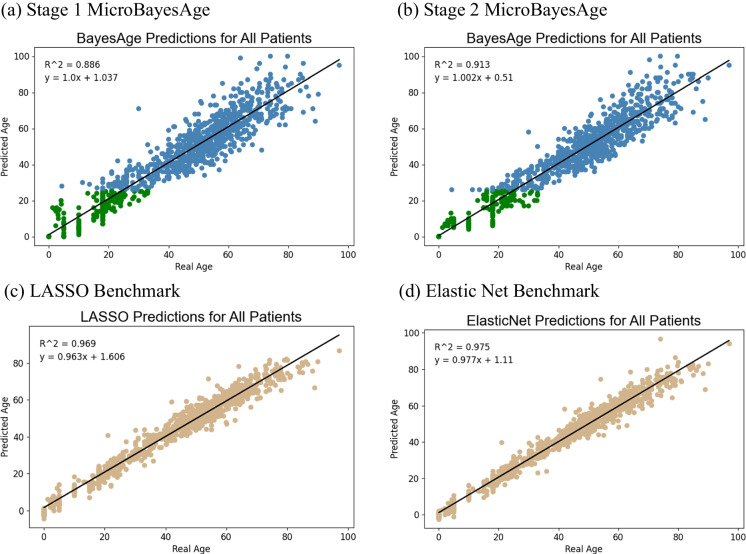
MicroBayesAge first and second stage age predictions plotted against real age for all patients. Age predictions older than 25 are shown in blue while age predictions of 25 or younger are shown in green. LASSO and elastic net age predictions are shown in tan for comparison. Trend lines are shown in black. ${R}^{2}$ metrics for each set of predictions are located in the upper-left corner of each sub-figure

The top 16 most correlated CpG sites for each of the two age cohorts were then selected, and LOWESS regression fits were calculated for both age cohorts (Fig. 3). Because separate subsets of patients were considered, a different set of 16 CpG sites were selected for each of the two age cohorts, and new regression fits were calculated. These newly calculated relationships were then used to generate the second stage of age predictions for the junior cohort and senior cohort in the validation dataset (Fig. 4).

**Fig. 5:**
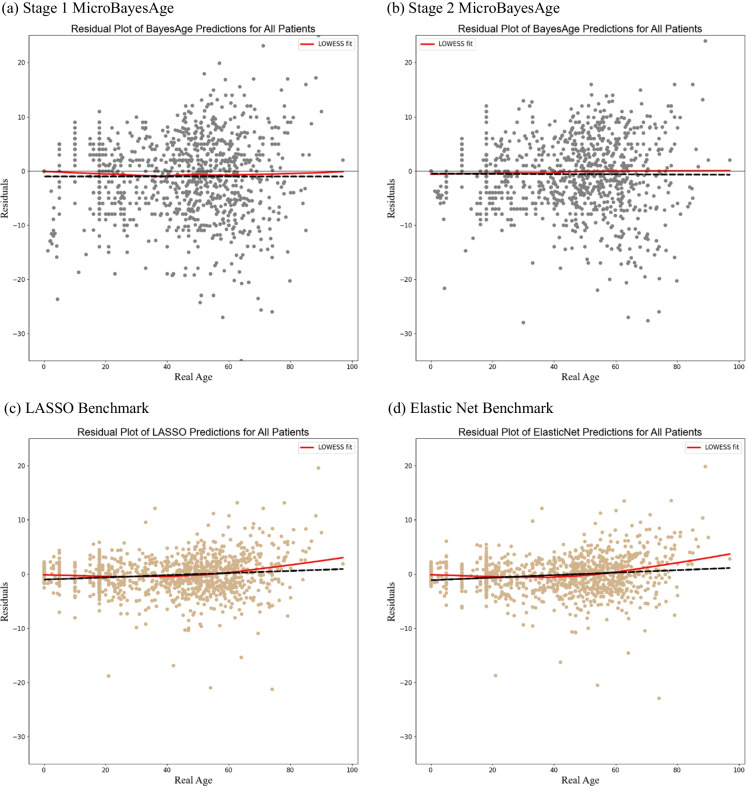
Residual plots of age predictions. MicroBayesAge first stage and second stage age predictions for all patients are shown in gray. LASSO and elastic net age predictions are shown in tan for comparison. Trend lines are shown in black and LOWESS fits are shown in red

In order to determine the bias of our predictions, we calculated the residuals that are the difference between our predicted ages and the real ages, for each of the testing samples (Fig. 5). We also calculated the deviance from the linear fit of the predicted ages for MicroBayesAge and our comparison models (Fig. S2). Despite its lower accuracy when compared to elastic net, the advantage that BayesAge exhibits over alternate methods of age prediction are its comparatively unbiased age predictions, meaning there is minimal correlation between the residuals and the real age of the samples. Both the first-stage and second-stage predictions of MicroBayesAge similarly exhibit relatively unbiased age predictions when compared to our LASSO and elastic net age predictions.

**Fig. 6:**
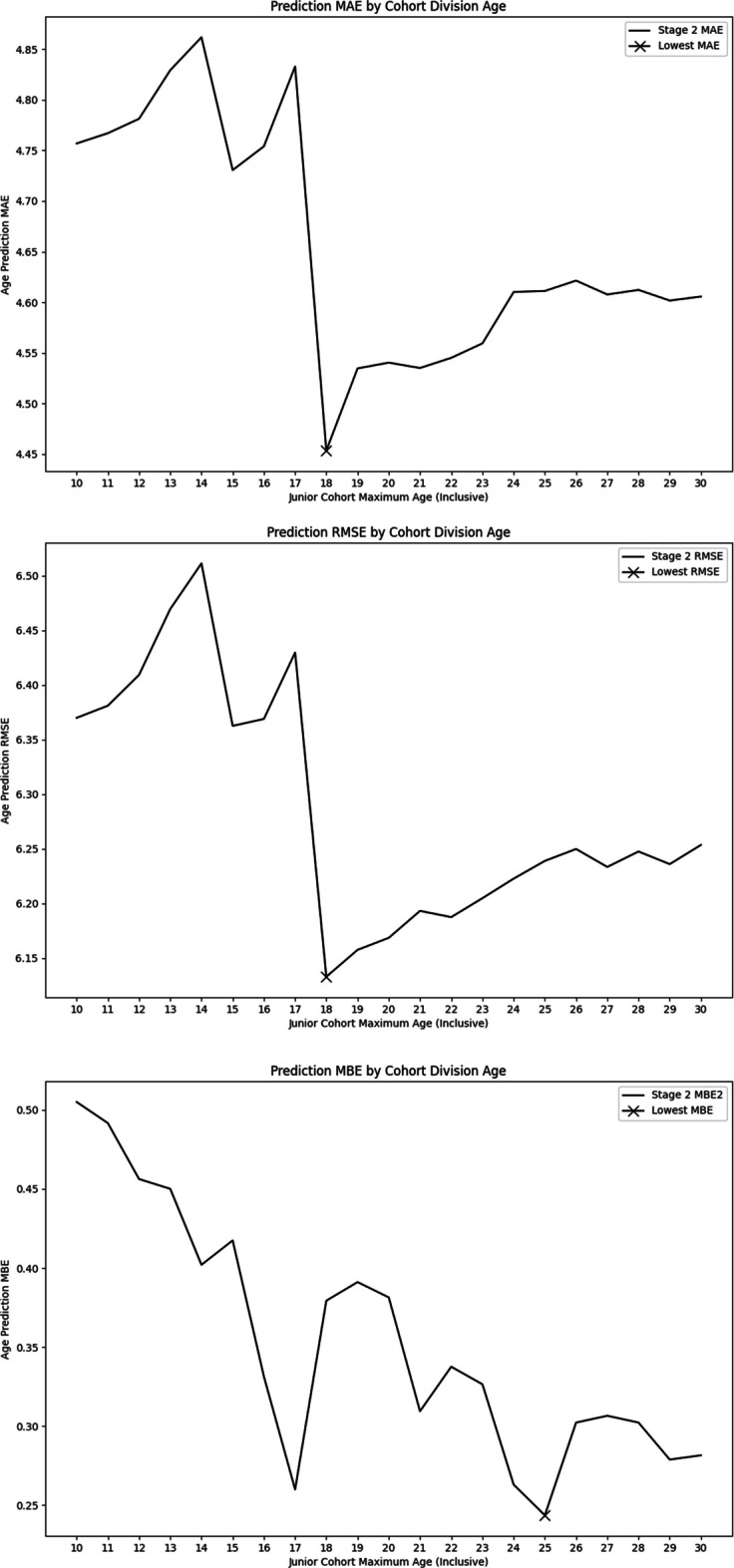
Comparison of overall MAE, RMSE, and MBE for the second stage age predictions for each tested age cutoff. The lowest value is marked with an X in all three graphs

To determine the optimal cutoff age, we selected the cutoff age with the lowest MBE, since the primary advantage of the BayesAge framework is its low bias compared to other models, rather than its absolute error. A cutoff age of 25 years was found to be optimal since it resulted in the lowest MBE. Utilizing this cutoff age reduced the MBE of the age predictions by 59.5% to 0.24 years, significantly lower than the first-stage MBE of 0.60 years (Fig. 6). After testing, we found that the addition of the second stage of our age prediction algorithm significantly decreased the MAE of the age predictions. This improvement in accuracy was primarily driven by a large decrease in the MAE of age predictions for the junior cohort, defined as 25 years old or younger. There was comparatively less change in the MAE of age predictions for the senior cohort of patients older than 25 years, with only a small increase in accuracy observed.

The second stage significantly improved the overall MAE and RMSE for patients of all ages. The MAE decreased by 10.6% from 5.16 to 4.61 years and the RMSE decreased by 8.5% from 6.82 to 6.24 years. This increase in the accuracy of the age predictions was primarily due to the improvement in accuracy for patients 25 years old or younger. For these patients, the MAE decreased by 25.4% from 1.24 to 0.93 years and the RMSE decreased by 19.7% from 2.95 to 2.37 years. There was comparatively less improvement in the accuracy of age predictions for patients older than 25 years. The MAE for these patients decreased by 5.9% from 3.92 years to 3.68 years and the RMSE decreased by 6.1% from 6.15 years to 5.77 years. For comparison, LASSO regression showed an MAE of 2.42 years and an RMSE of 3.39 for all patients while elastic net showed an MAE of 2.40 years and an RMSE of 3.37 years for all patients.

### Prediction by sex

We theorized that accounting for sex-based differences in the relationship between CpG site methylation and age could increase the accuracy of the MicroBayesAge predictions [39]. To test this, sex-specific datasets were created by splitting the training and testing datasets into male and female cohorts. Each contained a broad range of ages distributed similarly to those of the original full dataset (Fig. 1).

Within the sex-specific datasets, slight increases in the accuracy of age predictions were found for the male-specific model; however, no significant change was observed in the accuracy of age predictions for the female-specific model compared to the predictions of the mixed-sex model trained on all of the patients.

For the male-specific second-stage predictions, the overall MAE and RMSE for patients across all ages improved slightly in comparison to the mixed-sex second-stage predictions. The male-specific MAE was 4.37 years in comparison to 4.61 years for the mixed-sex MAE. The male-specific RMSE was 5.90 years in comparison to 6.24 years for the mixed-sex RMSE. These improvements were primarily driven by an increase in age prediction accuracy for the senior cohort. For patients 25 or younger, the second-stage male-specific MAE of 0.94 years and RMSE of 2.33 years were comparable to the mixed-sex MAE and RMSE. For patients older than 25, the sex-specific MAE of 3.43 years and RMSE of 5.42 years were lower than the mixed-sex MAE and RMSE.

For the female-specific second-stage predictions, the overall MAE was 4.76 years, and the RMSE was 6.39 years for patients of all ages, which was not a significant change compared to the mixed-sex MAE of 4.63 years and RMSE of 6.24 years. The MAE and RMSE for the sex-specific junior cohort and senior cohort were similarly comparable for female patients to those of the mixed-sex age predictions.

## Discussion

We developed a new version of the BayesAge framework, which uses microarray data to generate accurate age predictions by analyzing CpG site methylation levels. Like previously developed implementations of the BayesAge framework, we found that the primary advantage of MicroBayesAge over other commonly used epigenetic clocks is its low age prediction bias.

MicroBayesAge only predicts chronological age. It does not attempt to predict biological age, defined as the progressive decline in health and increase in morbidity which occurs over time. Previous research has shown that different individuals can experience substantially different rates of biological aging, including age-associated changes in DNA methylation, relative to their chronological aging [40]. MicroBayesAge solely utilizes a 16 CpG site subset of DNA methylome data to predict chronological age, so it exhibits inherent limitations in prediction accuracy when compared to other epigenetic clocks which focus more on biological aging [41], utilize a larger number of CpG sites for age prediction [42], or consider additional age-associated data to make predictions [6].

We further acknowledge the limitations of using same-age samples as technical replicates, particularly with our dataset, which includes a very high number of samples specifically aged 18 years old. We tested data division into bins of equal sample size to estimate variance, but found that prediction accuracy slightly decreased. In addition, we attempted smoothing variance across age rather than methylation level for each CpG site, which produced equally accurate results. Future studies might consider additional exploration into the patterns of variance in methylation values by including more replicates, particularly in the teen and senior age groups, for more robust variance estimation.

To achieve higher accuracy, MicroBayesAge leverages the differences in the relationship between methylation and age across different age groups. Using a two-stage process for both training and prediction, MicroBayesAge is able to account for these variations between age cohorts when constructing prediction algorithms and categorizes data with unknown ages via initial rough estimates before generating more precise predictions. This subdivision of input data into cohorts based on estimated age allows MicroBayesAge to apply specifically tailored analyses to each age cohort individually. We tested a range of possible age cutoffs to subdivide the cohorts and selected 25 as the optimal cutoff age based upon its MBE.

The second stage of MicroBayesAge shows a significantly improved overall age prediction accuracy compared to the first stage. Notably, it achieves much greater accuracy in predicting ages for the junior cohort, but shows comparatively less dramatic improvements in the accuracy of the age predictions for the senior cohort. The relationship between methylation and real age for all of the most correlated CpG sites is nearly linear for the senior cohort, with the LOWESS fit of methylation vs age closely matching the linear fit. In contrast, the relationship between methylation and real age for all of the most correlated CpG sites is nonlinear for the junior cohort, with the LOWESS fit of methylation vs. age diverging significantly from the linear fit. In addition, the residuals of the age predictions indicate minimal bias for both the first and the second stage age predictions generated by MicroBayesAge when compared to the predictions produced by the LASSO and elastic net benchmarks.

Many epigenetic clocks assume that the relationship between DNA methylation and chronological age is linear when making age predictions. This has been found to be a fairly accurate assumption for humans in adulthood. However, previous research has suggested that the relationship between DNA methylation and chronological age is nonlinear during childhood development and adolescence, and may also be nonlinear for elderly people aged 80 + years old [43]. The nonlinearity of this relationship during childhood and adolescence would explain the increase in accuracy of our model for the junior cohort, as the BayesAge framework is optimized for modeling nonlinear relationships. This could also explain the comparative lack of improvement in prediction accuracy for the senior cohort, which would primarily include samples exhibiting a linear relationship that our framework lacks advantages in modeling. It may be possible to improve the model by partitioning the senior cohort further into a 25- to 79-year-old cohort and an 80 + year-old cohort in order to better model the predicted nonlinearity of the age-associated methylation in the 80 + cohort.

We also considered the possibility that male and female patients might exhibit differing relationships between age and CpG site methylation. To test this, we split the patients in the training and testing datasets into two groups based on sex. The MicroBayesAge framework was then trained and tested on male and female patients separately to determine if accuracy would improve with sex-specific training and age prediction. We found slight improvements in age prediction accuracy for male patients when compared to the mixed-sex predictions, but no change in accuracy for female patients.

Past research has found that the optimal cutoff age of juvenile development is significantly higher for females than for males, with the optimal male cutoff age being approximately 20 years and the optimal female cutoff age being approximately 30 years. These differences in optimal cutoff age likely explain the lack of improvements in accuracy for the female sex-specific age predictions as compared to the male. While 25 years is equally near to both sex-specific optimal cutoff ages, previous research suggests that accuracy declines more rapidly as the cutoff diverges from optimal for females than for males [43]. Given this, repeating the cutoff age range analysis for both sexes separately and implementing differing cutoff ages for males and females might pose another means of further improving overall age prediction accuracy for the sex-specific models.

In conclusion, MicroBayesAge uses the partitioning of training datasets and input data to achieve greater accuracy. By separating broad datasets into more specific groups, it can better account for differences in epigenetic aging between different classes of patients. This strategy has potential for further expansion. Age-based partitioning could be further optimized to generate more refined predictions, and further means of subdividing data based on other criteria could potentially provide further improvements in accuracy. For instance, it could improve accuracy further if patients were subdivided into a greater number of smaller, more focused age cohorts. Additional data points, such as information regarding chronic medical conditions, might also be used to subdivide datasets and potentially produce more accurate predictions.

## Supplementary information

Below is the link to the electronic supplementary material.

ESM 1 (DOCX 2.21 MB)

## Data Availability

The code and data used for this study are available at https://github.com/nicolecnolan/MicroBayesAge.
