# Supplementary material for: MicroBayesAge: a maximum likelihood approach to predict epigenetic age using microarray data
Source: GeroScience. 2025 May 31;48(1):691–704. doi: 10.1007/s11357-025-01716-4 (PMC12972212; doi:10.1007/s11357-025-01716-4)
Supplement: Supplementary file 1 — (DOCX 2.21 MB) [file 11357_2025_1716_MOESM1_ESM.docx]

**MicroBayesAge: A Maximum Likelihood Approach to Predict Epigenetic Age Using Microarray Data Supplemental**

**Nicole Nolan^†^ | Megan Mitchell^†^ | Lajoyce Mboning | Louis-S. Bouchard | Matteo Pellegrini**

April 2025


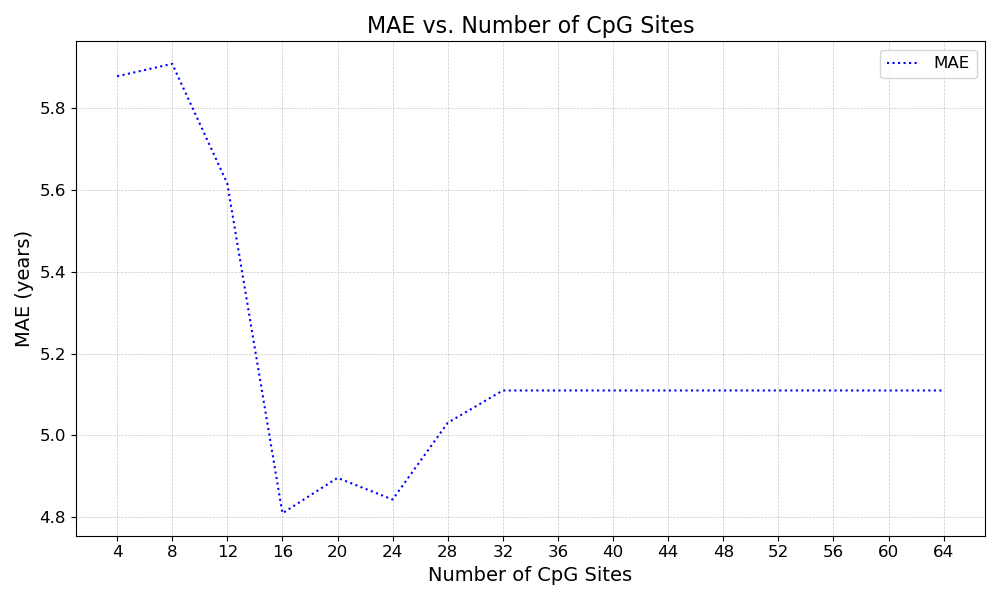


**Fig. S1** Plot of average mean absolute error vs. number of CpG sites. The minimum error was achieved using only the top 16 most highly Spearman correlated sites.

(a) Stage 1 MicroBayesAge (b) Stage 2 MicroBayesAge


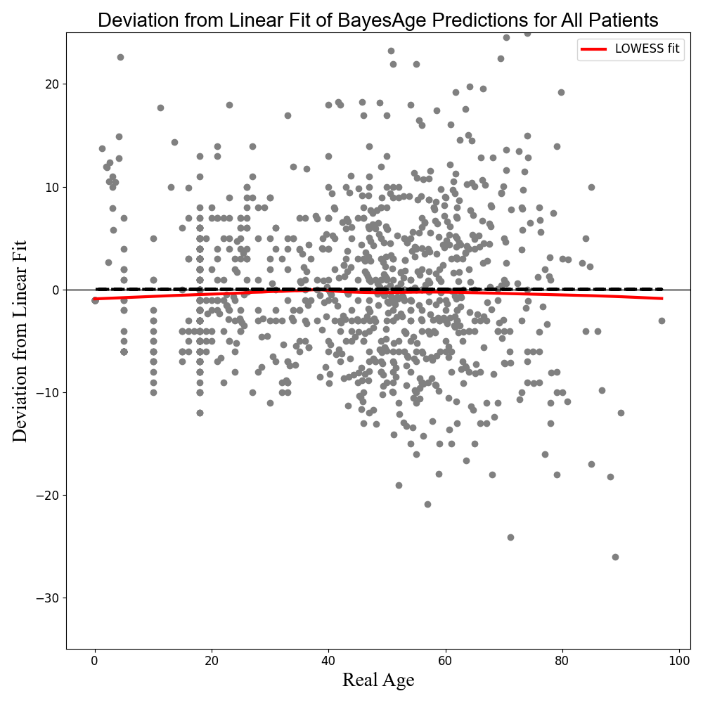

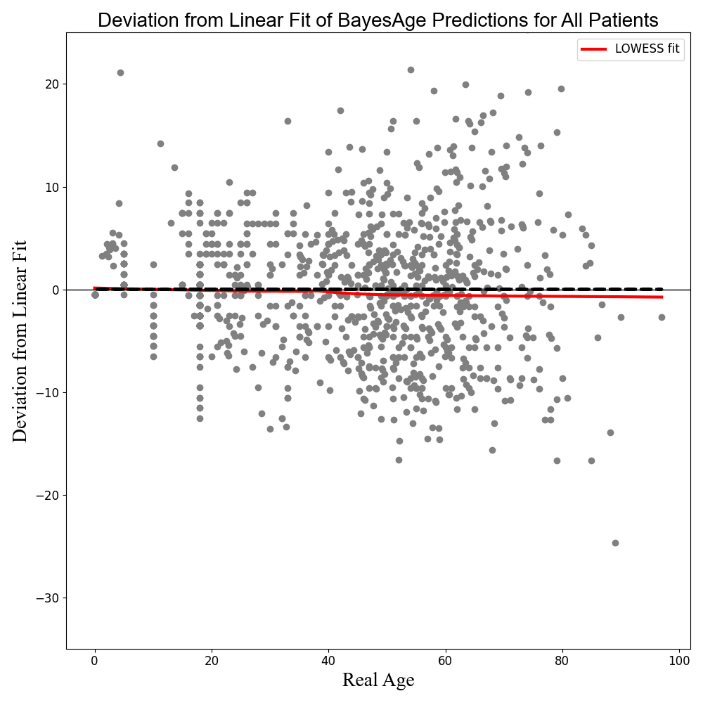


(c) LASSO Benchmark (d) Elastic Net Benchmark


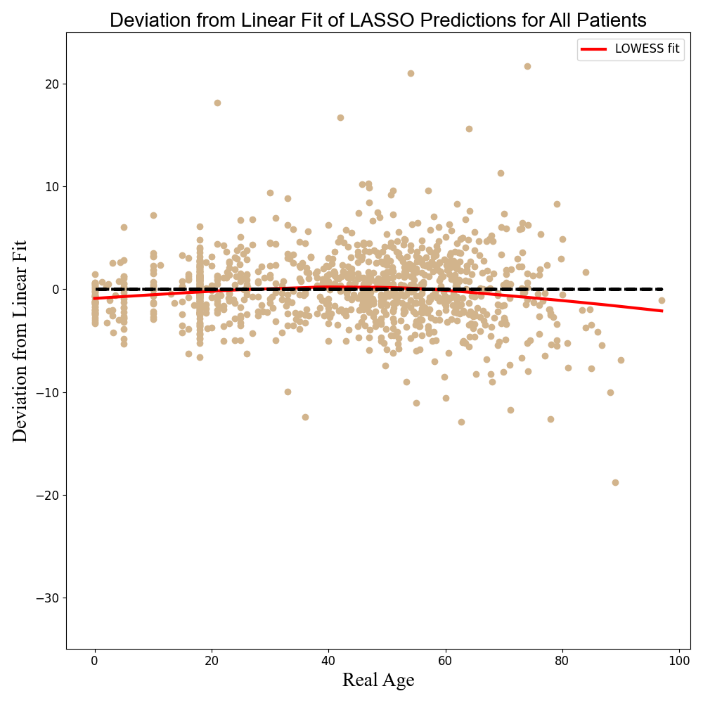

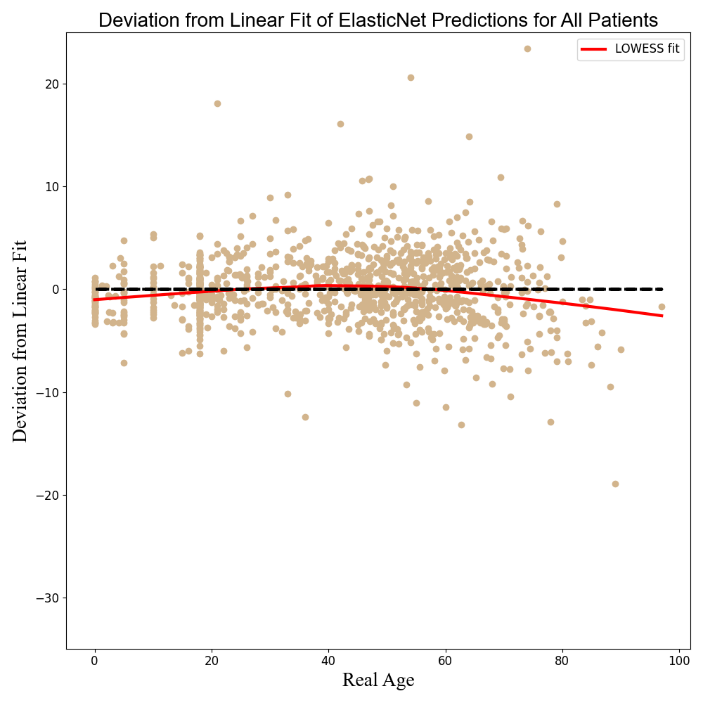


**Fig. S2** Residual plots of age predictions showing deviation from linear fit. MicroBayesAge first stage and second stage age predictions for all patients are shown in gray. LASSO and ElasticNet age predictions are shown in tan for comparison. Trend lines are shown in black and LOWESS fits are shown in red.


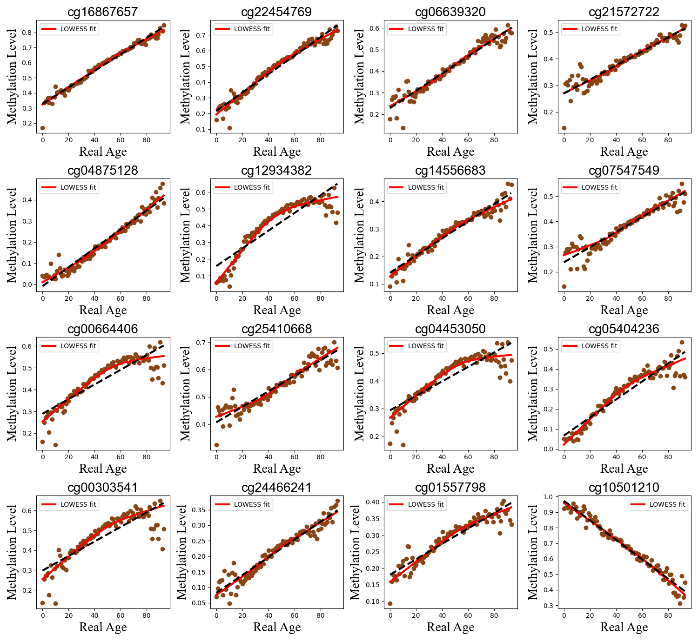


(a) All male samples


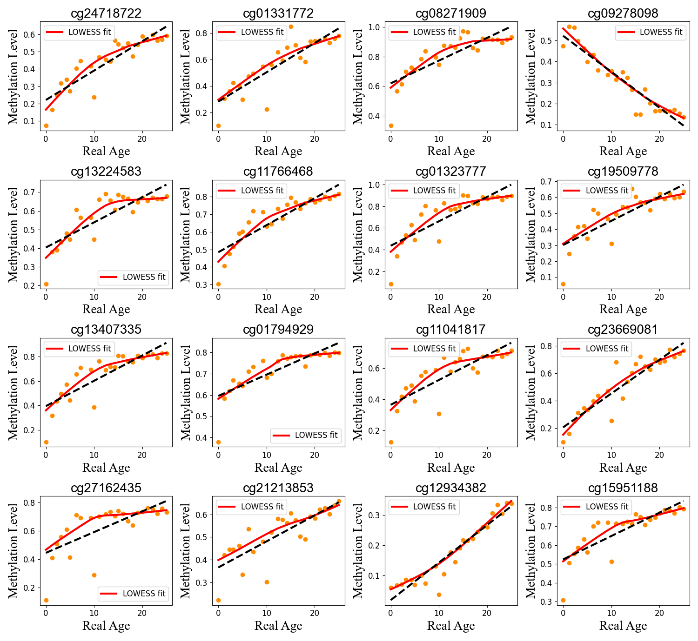

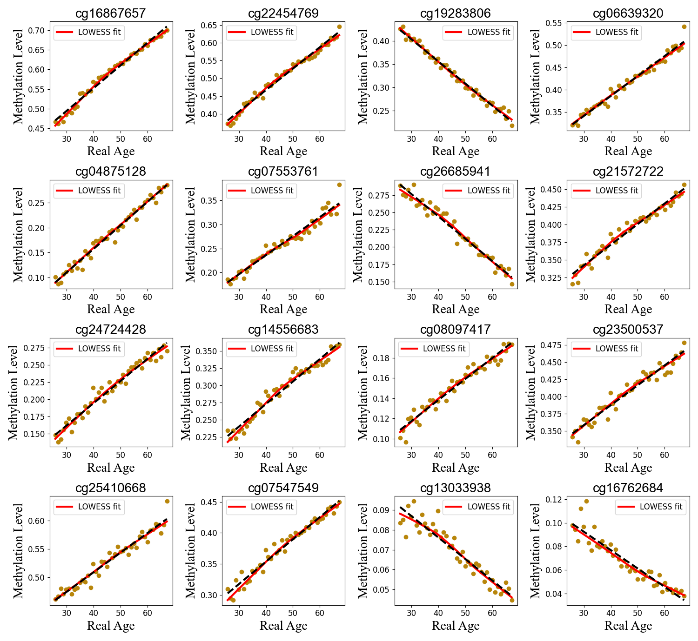


(b) Male junior cohort (c) Male senior cohort

**Fig. S3** Comparison of LOWESS regression fits, shown in red, with $\tau$ of 0.7 of the relationship between methylation and age for the top 16 most correlated CpG sites. Scatter points are shown in (a) brown for all male patients, (b) in orange for the male junior cohort, and (c) in yellow for the male senior cohort.


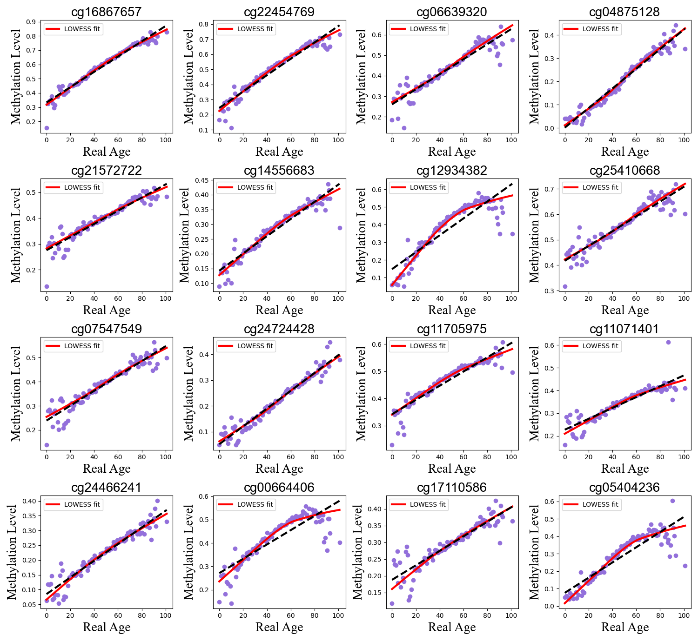


(a) All female samples


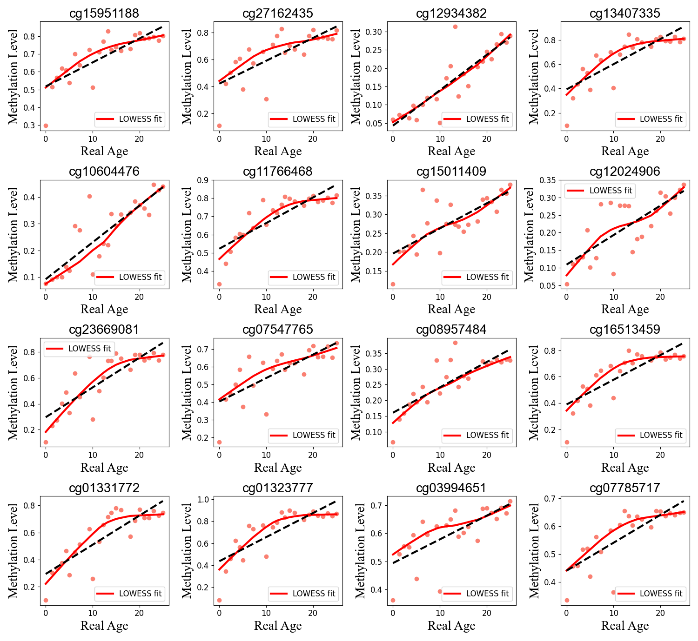

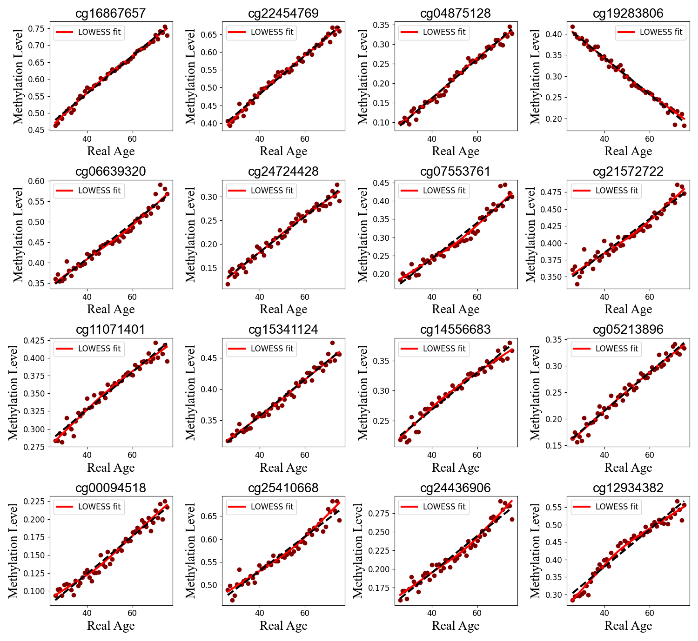


(b) Female junior cohort (c) Female senior cohort

**Fig. S4** Comparison of LOWESS regression fits, shown in red, with $\tau$ of 0.7 of the relationship between methylation and age for the top 16 most correlated CpG sites. Scatter points are shown in (a) purple for all female patients, (b) in salmon for the female junior cohort, and (c) in dark red for the female senior cohort.

(a) Stage 1 MicroBayesAge (b) Stage 2 MicroBayesAge


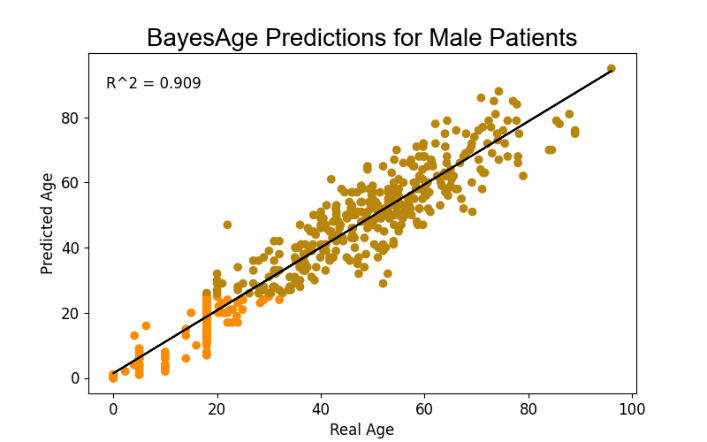

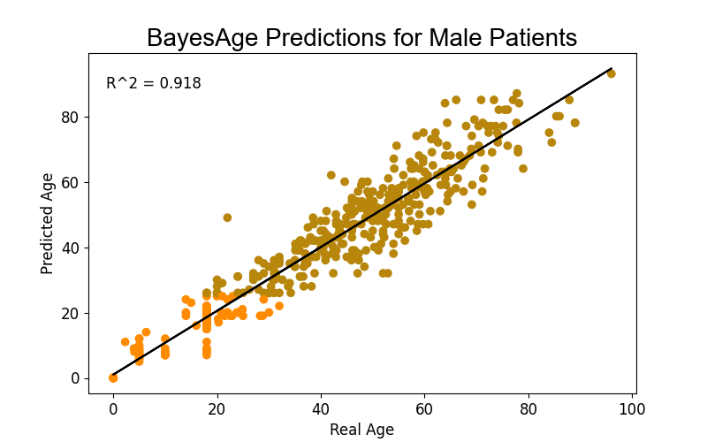


(c) LASSO Benchmark (d) Elastic Net Benchmark


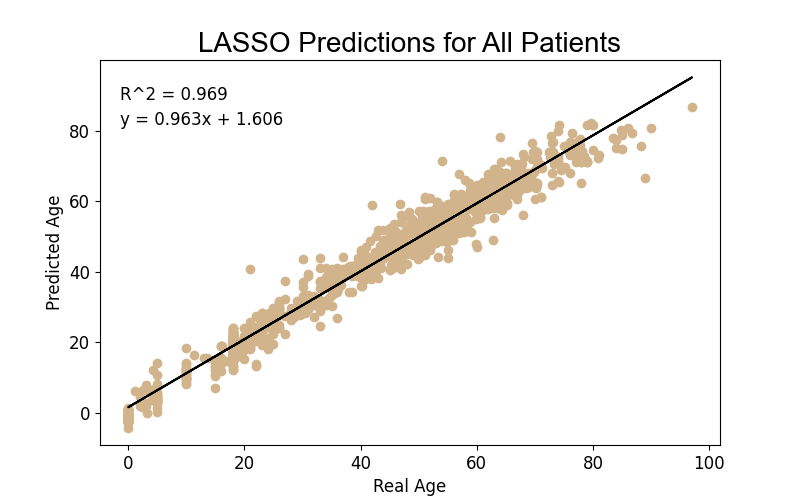

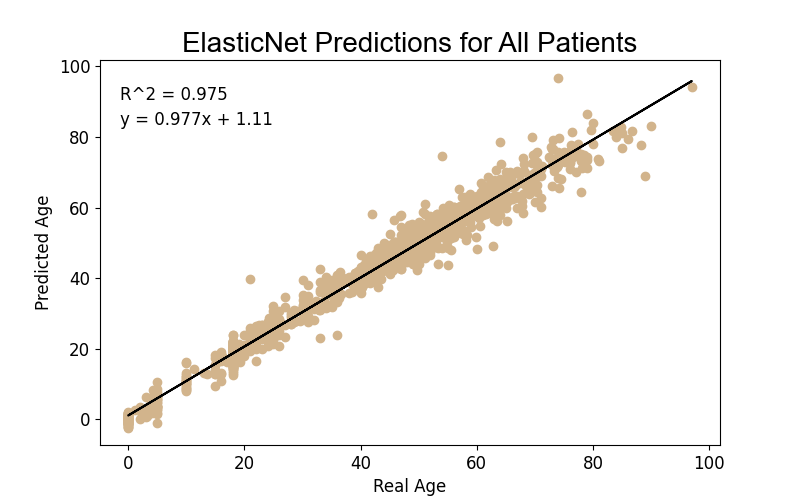


**Fig. S5** MicroBayesAge first and second stage age predictions plotted against real age for all male patients. Age predictions older than 25 are shown in yellow while age predictions of 25 or younger are shown in orange. LASSO and ElasticNet age predictions are shown in tan for comparison. Trend lines are shown in black. $R^{2}$ metrics for each set of predictions are located in the upper-left corner of each sub-figure.

(a) Stage 1 MicroBayesAge (b) Stage 2 MicroBayesAge


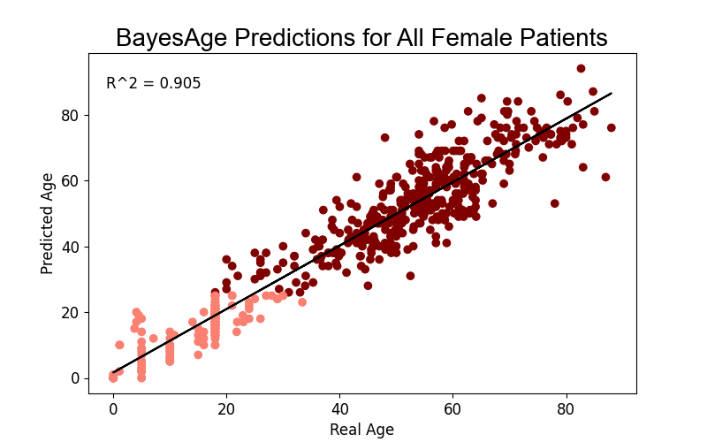

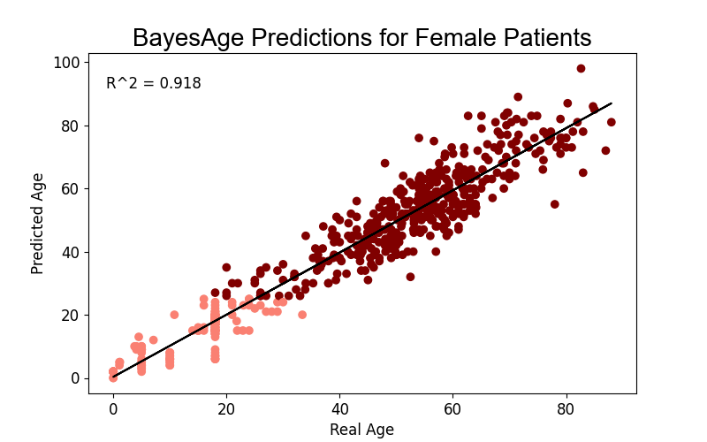


(c) LASSO Benchmark (d) Elastic Net Benchmark


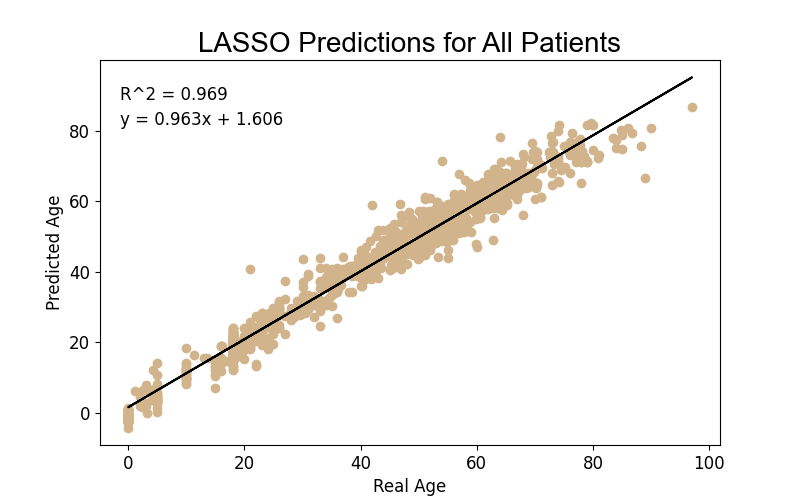

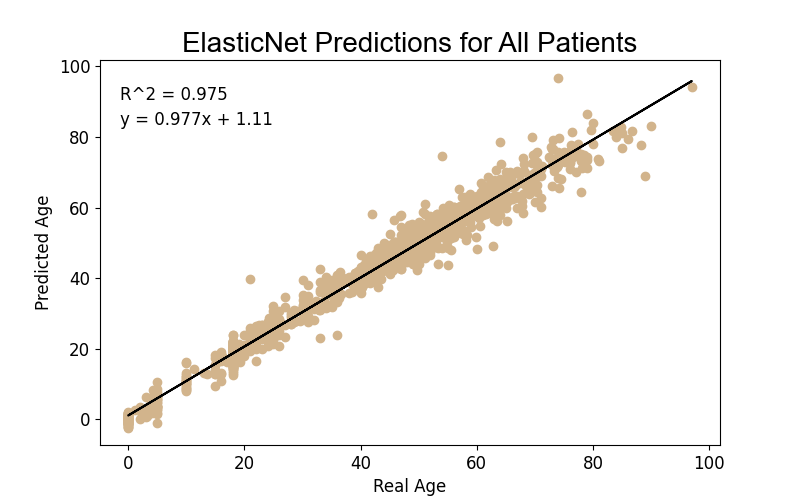


**Fig. S6** MicroBayesAge first and second stage age predictions plotted against real age for all female patients. Age predictions older than 25 are shown in dark red while age predictions of 25 or younger are shown in salmon. LASSO and ElasticNet age predictions are shown in tan for comparison. Trend lines are shown in black. $R^{2}$ metrics for each set of predictions are located in the upper-left corner of each sub-figure.

(a) Stage 1 MicroBayesAge (b) Stage 2 MicroBayesAge


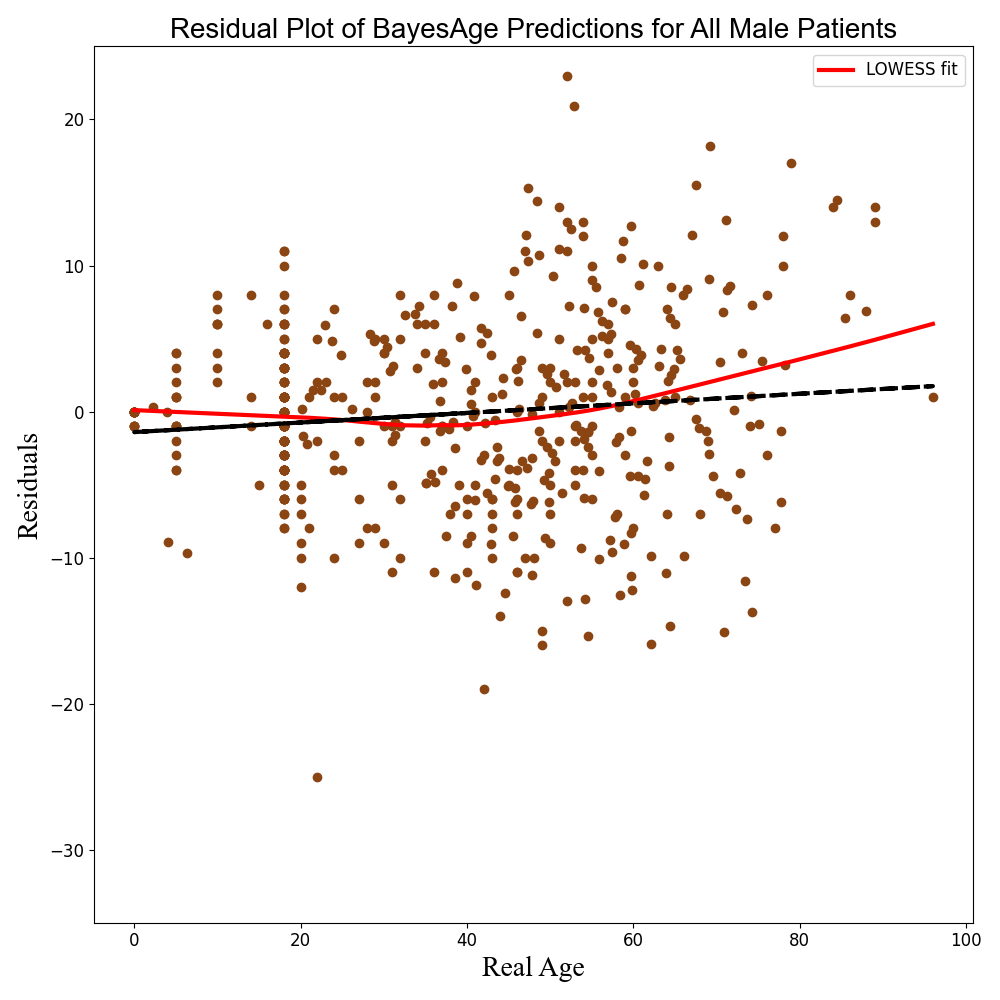

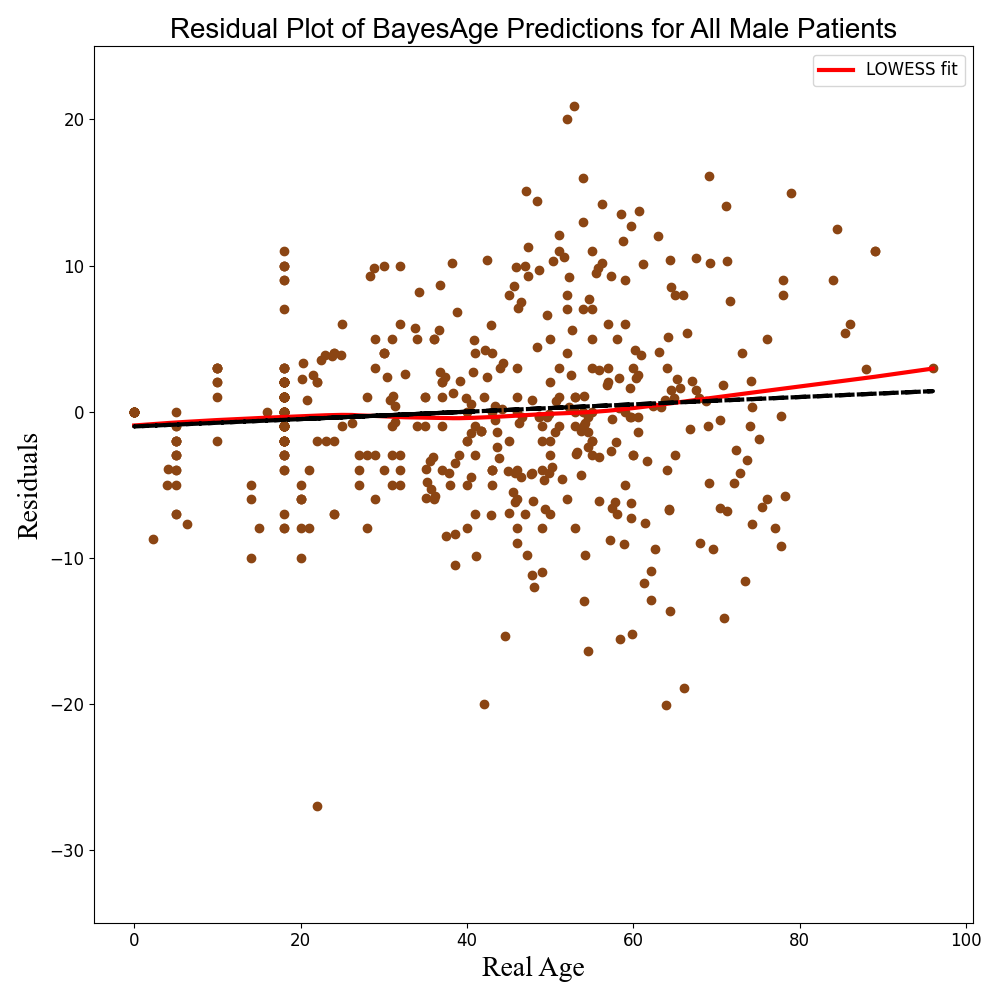


(c) LASSO Benchmark (d) Elastic Net Benchmark


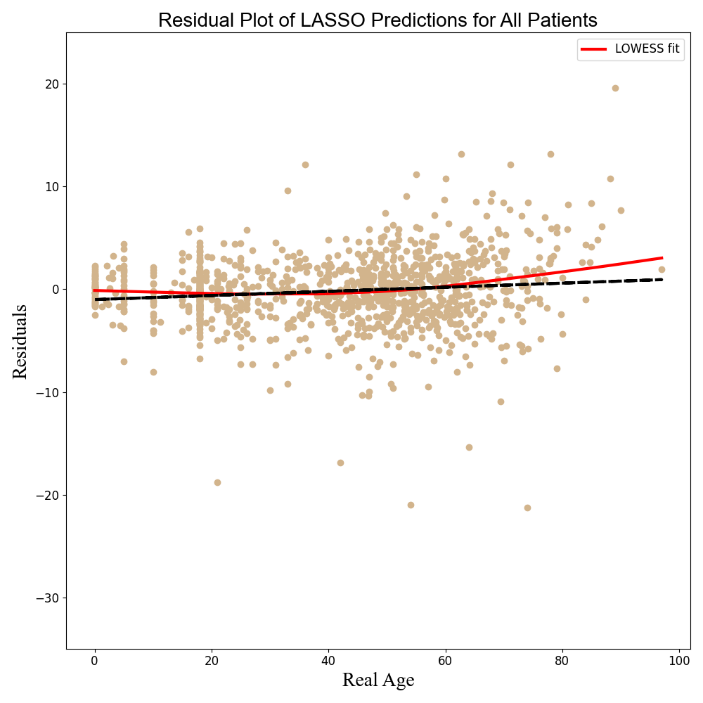

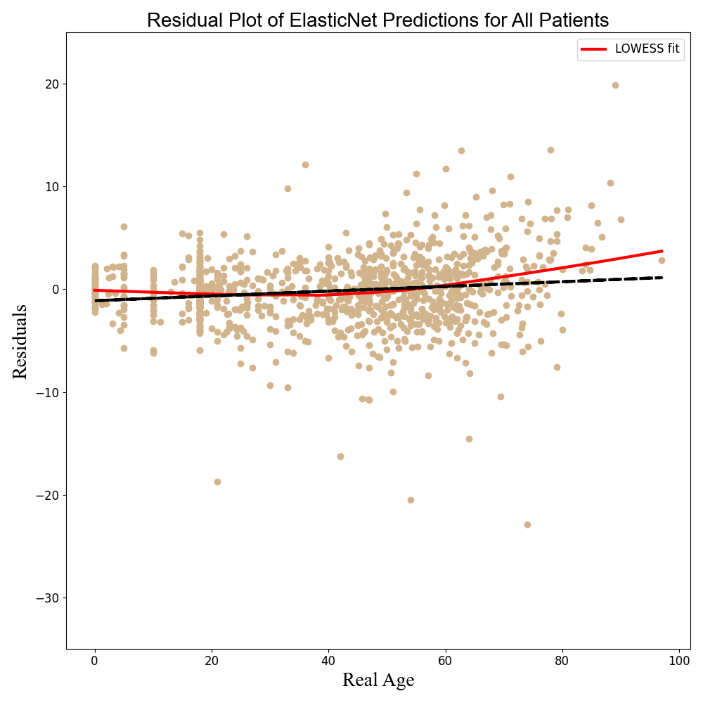


**Fig. S7** Residual plots of age predictions. MicroBayesAge first stage and second stage age predictions for all male patients are shown in brown. LASSO and ElasticNet age predictions are shown in tan for comparison. Trend lines are shown in black and LOWESS fits are shown in red.

(a) Stage 1 MicroBayesAge (b) Stage 2 MicroBayesAge


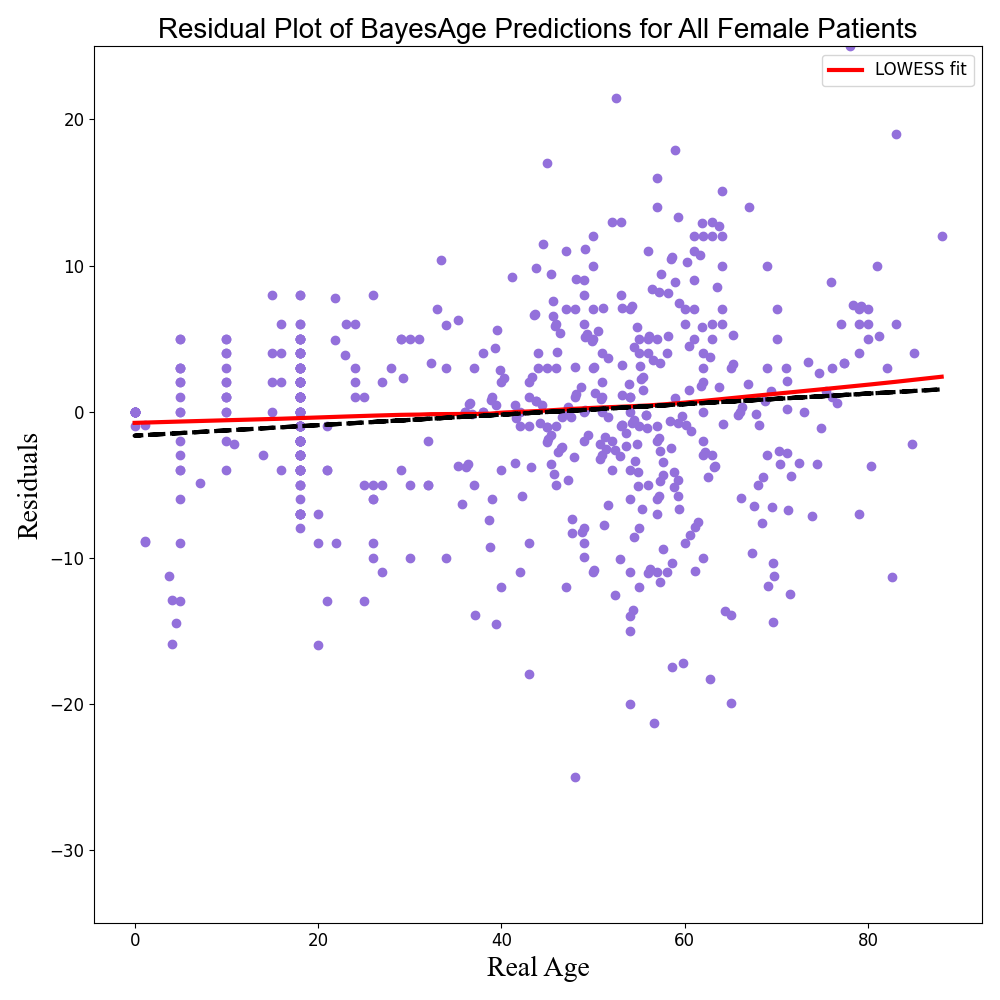

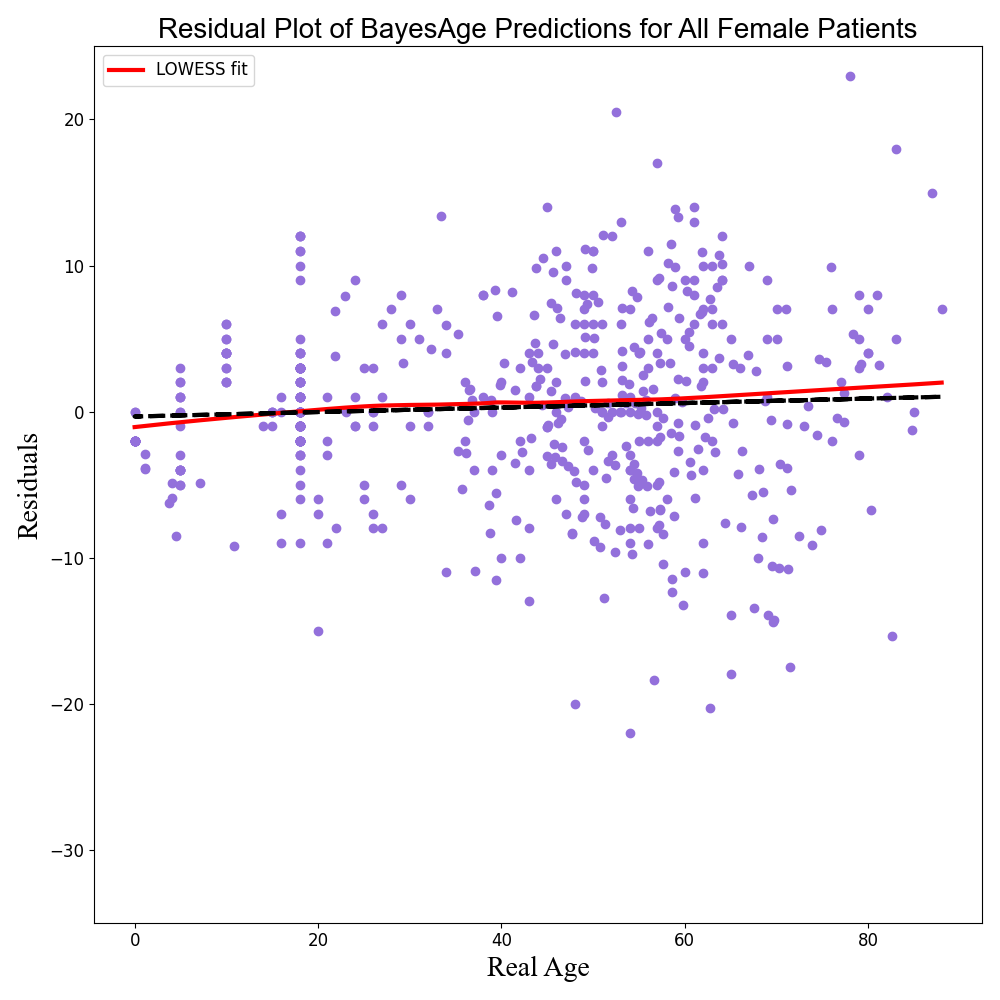


(c) LASSO Benchmark (d) Elastic Net Benchmark


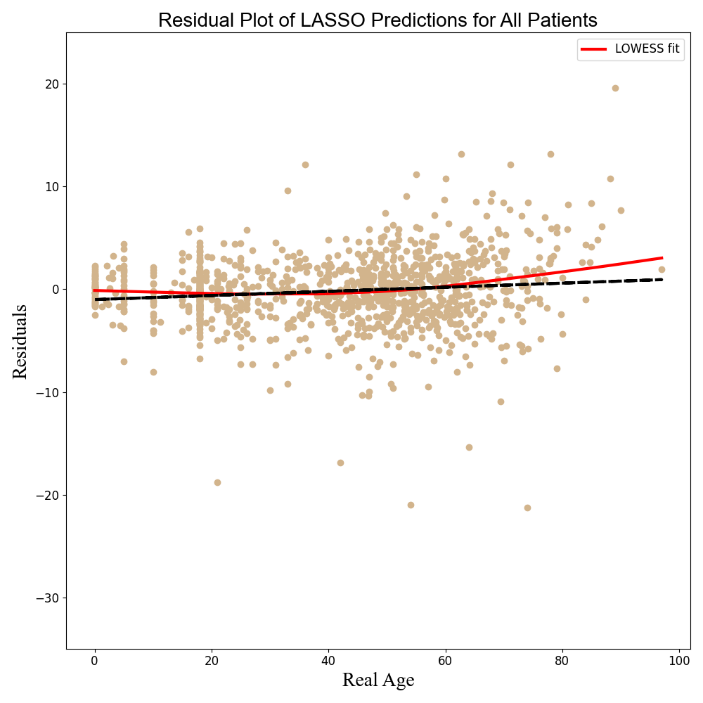

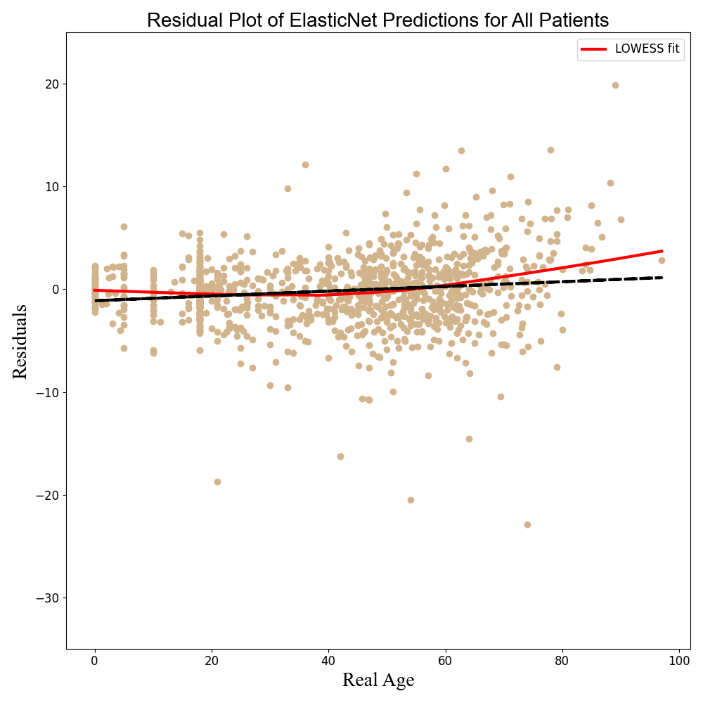


**Fig. S8** Residual plots of age predictions. MicroBayesAge first stage and second stage age predictions for all female patients are shown in purple. LASSO and ElasticNet age predictions are shown in tan for comparison. Trend lines are shown in black and LOWESS fits are shown in red.

(a) Stage 1 MicroBayesAge (b) Stage 2 MicroBayesAge


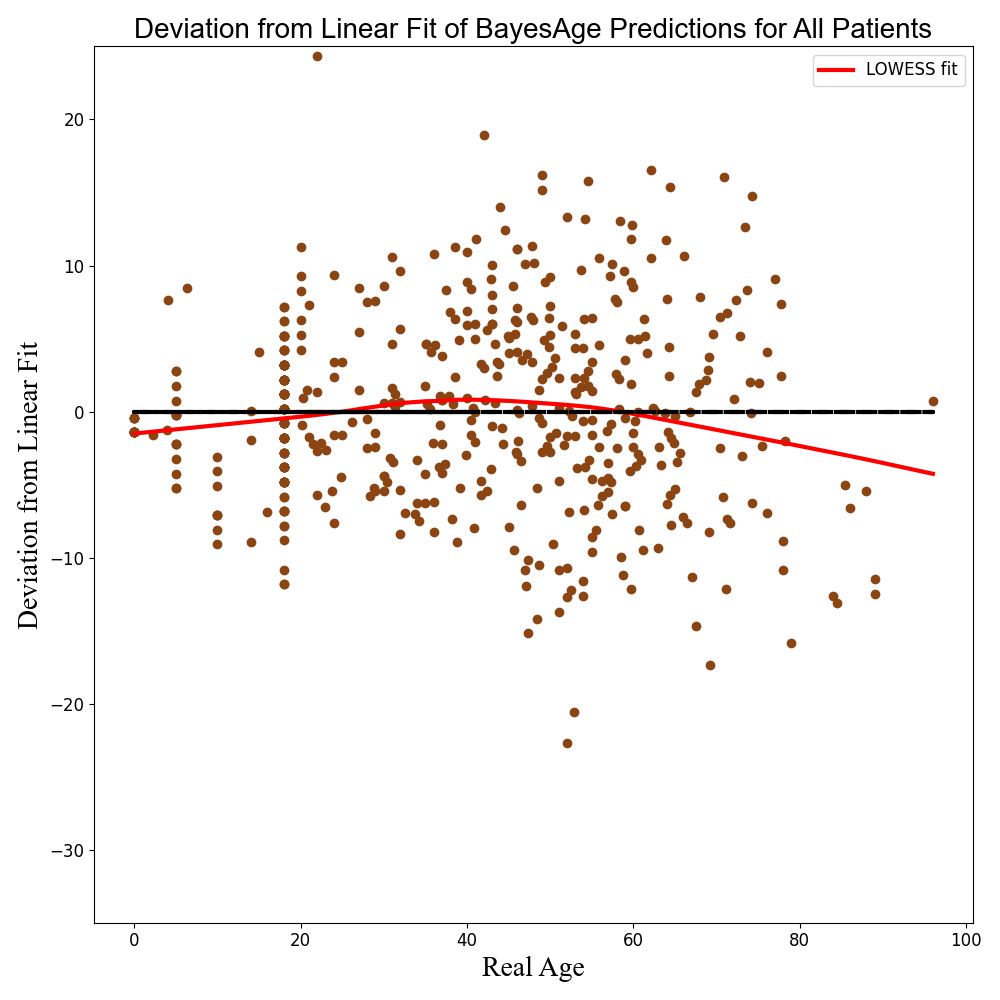

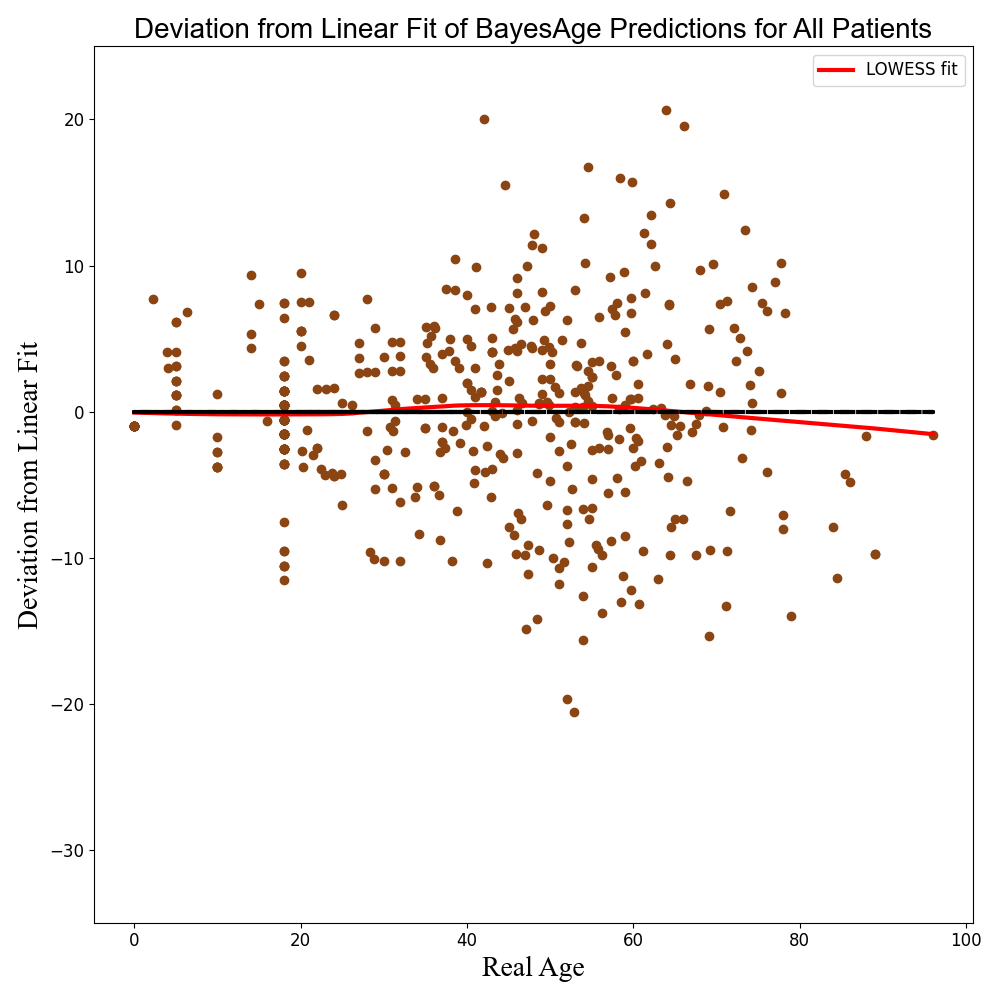


(c) LASSO Benchmark (d) Elastic Net Benchmark


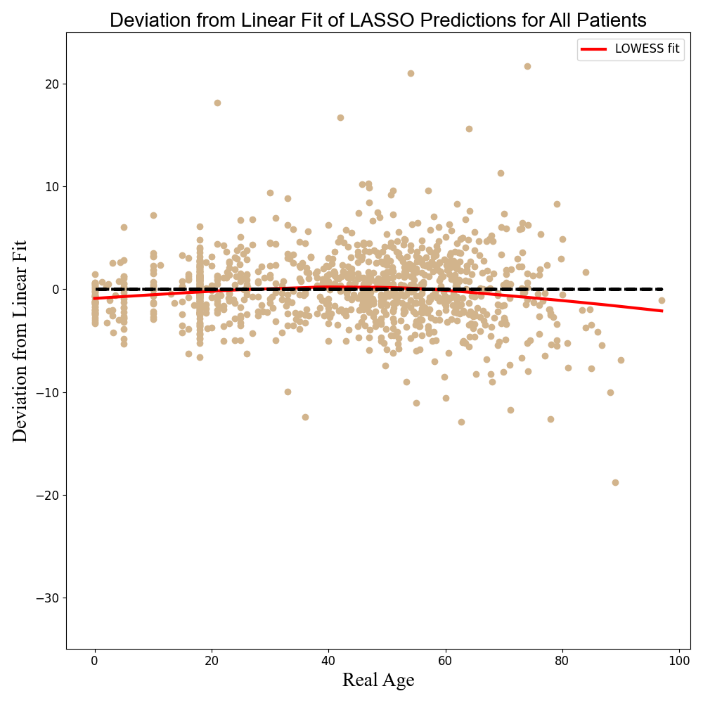

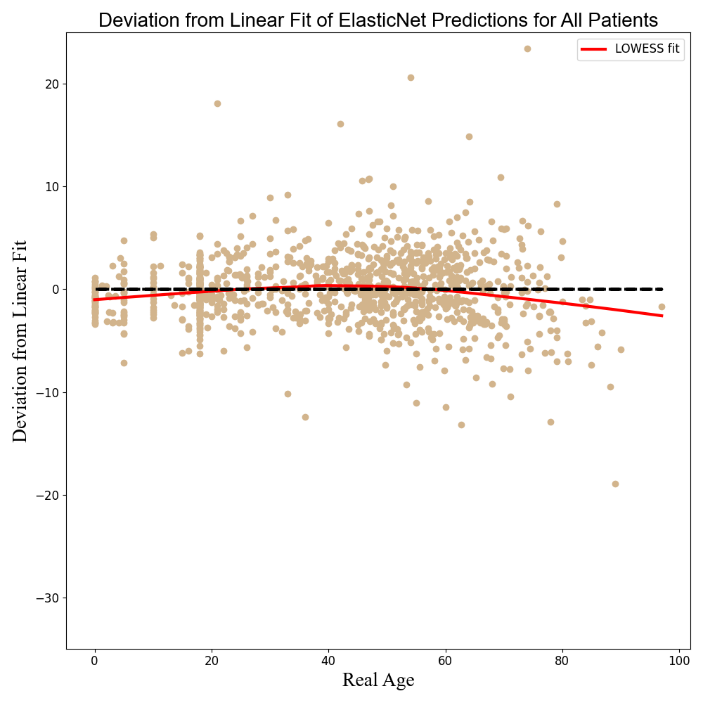


**Fig. S9** Residual plots of age predictions showing deviation from linear fit. MicroBayesAge first stage and second stage age predictions for all male patients are shown in brown. LASSO and ElasticNet age predictions are shown in tan for comparison. Trend lines are shown in black and LOWESS fits are shown in red.

(a) Stage 1 MicroBayesAge (b) Stage 2 MicroBayesAge


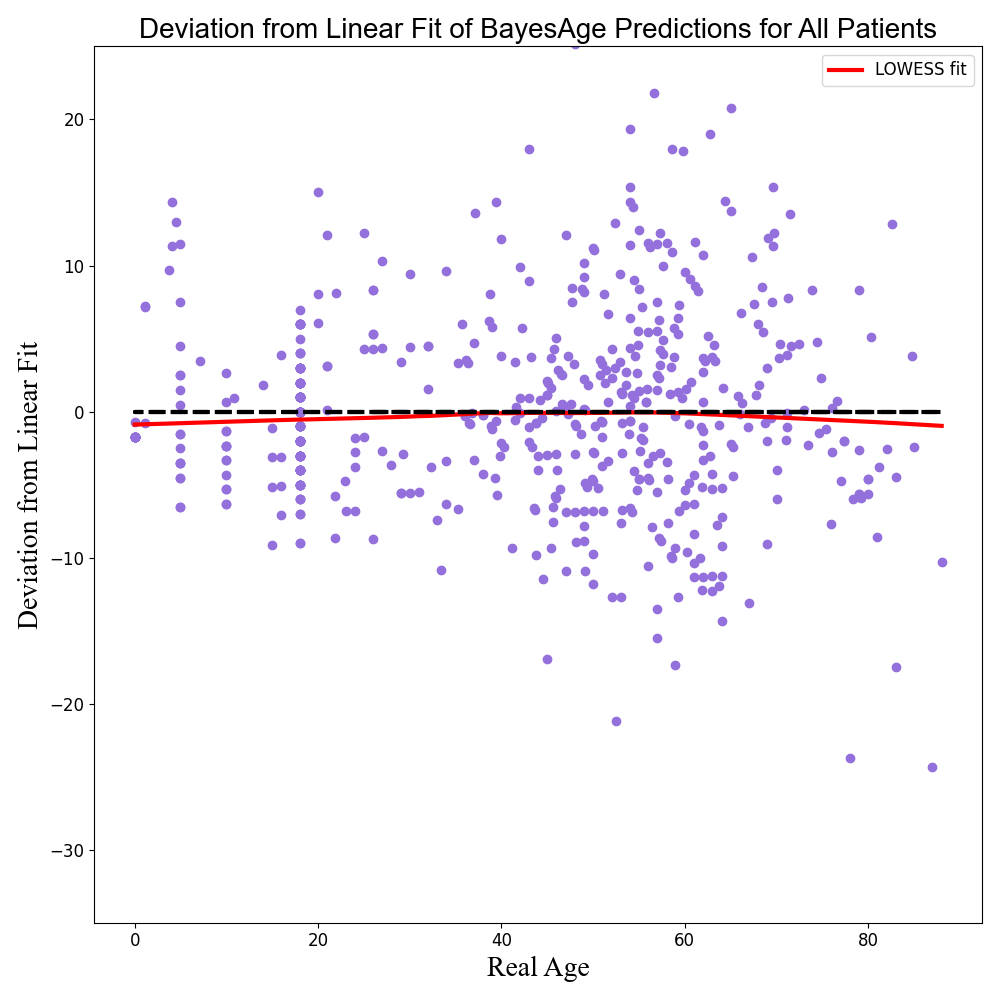

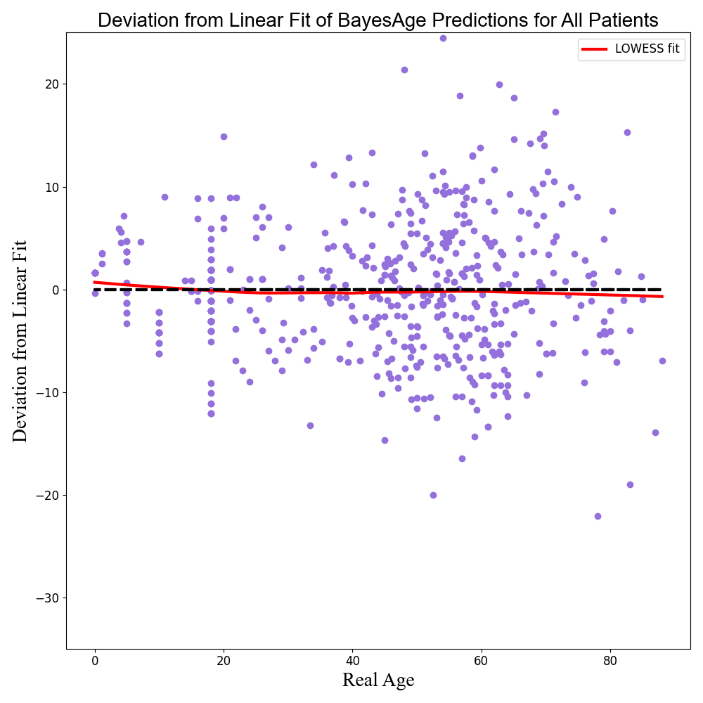


(c) LASSO Benchmark (d) Elastic Net Benchmark


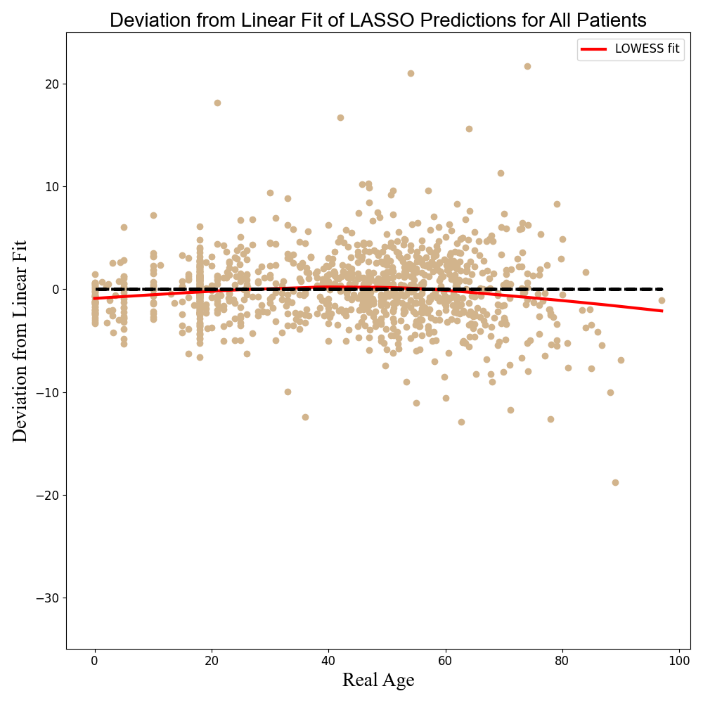

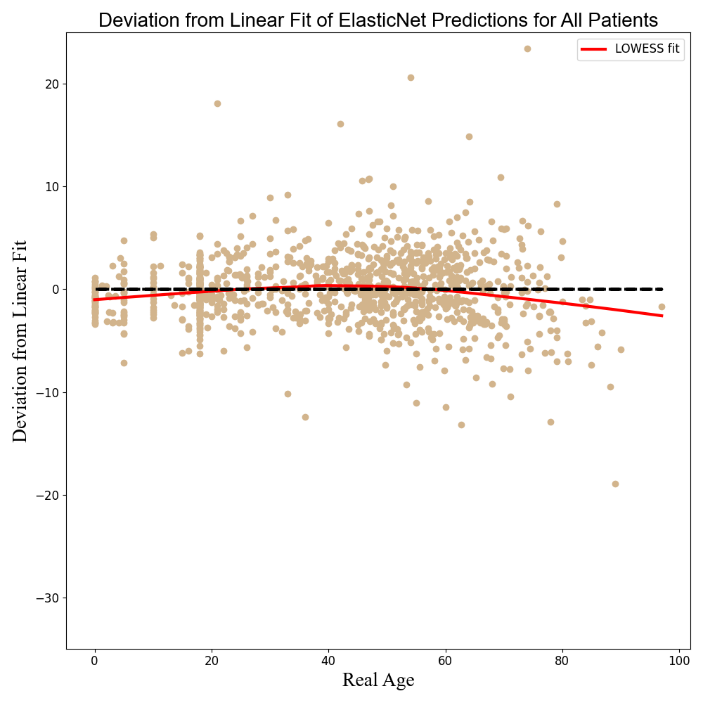


**Fig. S10** Residual plots of age predictions showing deviation from linear fit. MicroBayesAge first stage and second stage age predictions for all female patients are shown in purple. LASSO and ElasticNet age predictions are shown in tan for comparison. Trend lines are shown in black and LOWESS fits are shown in red.
